# Cases of Fever with Remarks

**Published:** 1846-01

**Authors:** J. R. Buck

**Affiliations:** Lecturer on the Theory and Practice of Medicine in the Louisville Summer School of Medicine; Louisville


### WESTERN JOURNAL

OF

### MEDICINE AND SURGERY.

JANUARY, 1846.

**Art. I.**—*Cases of Fever with remarks.* By J. R. Buck, M.D., Lecturer on the Theory and Practice of Medicine in the Louisville Summer School of Medicine.

D. K., æt. 27 years, very robust, strictly temperate, ship carpenter; health perfect till the fall of 1841, when he first came to Louisville, since which time he has had several attacks of mild intermittent fever. In May, 1844, I attended him several days during a slight attack of bilious fever. In July following I again prescribed for him for chills and fever.

On the 14th of August, 1844, was called to see him; attack commenced two days previous with alternate flashes of heat and chilliness continuing all day. 13th. Had slight but distinct chill, followed by considerable fever. The morning of the 14th I found him with slight fever; dull headache; anorexia; no nausea or vomiting; not the slightest pain or tenderness on pressure over any part of the abdomen; pulse rather frequent, and compressible. Bowels loose from Lee's pills taken on the 12th. Tongue coated with a dense brownish fur. ℞ Calomel and Dover's powder.

15th.—8 o'clock, A.M. More fever, with much more tenseness and fulness of pulse. Bled to 16 ounces. Calomel and Dover's powder repeated.

16th. No fever; in all respects better; some little tympanitis; bowels still too loose. ℞ Quinine grs. x, opii gr. i, to be taken at one dose; after which was very comfortable until 12 o'clock at night, when fever with delirium came on; frequent copious watery dejections during the night; no tenderness over the abdomen.

17th. Dr. Gross called in consultation. Blisters covering the abdomen; calomel and quinine ää grs. v ,morphiæ acetat. grs. ii, to be repeated every four hours, until the watery dejections ceased. The only effect of this prescription, which was repeated several times, was to produce free bilious evacuations, still, however, very watery. No sudamina. No petechiæ, or rose colored spots upon any part of the body. No sordes. Tongue became red and clean; sense of hearing impaired.

Died on the 20th; constant noisy delirium for 18 hours previous.

*Examination seven hours after death.*—Rigidity of limbs; no unnatural appearance of the body. *Brain* not examined. *Chest*, perfectly healthy throughout. *Abdomen*, very slight inflammatory redness in cardiac extremity of stomach; also same in duodenum. *Jejunum* healthy. In lower part of ilium seven patches of Peyer's glands ulcerated, with slight inflammation of the mucous membrane between them. *Cæcum* larger than natural; whole internal lining membrane in a high state of inflammation, with numerous patches of a greenish yellow lymph upon it. *Colon* healthy. *Rectum*, intense inflammation, with some greenish yellow lymph.—*Spleen* two-thirds larger than natural, and much softened. *Liver* and *kidneys* healthy.

Case II.—J. H., æt. 20, ship carpenter, stout, robust, habts good, generally healthy. In July, 1844, had erysipelas of face and neck, mild, yielding in a few days to laxatives, with argen. nitrat. as an external application.

August 31st, 1844. Called to see him; had been slightly indisposed for two days; some fever; very slight pain in the lower part of the abdomen upon firm pressure; tongue covered with a brownish coat; pulse 88, moderately full; three dejections in 15 hours, thin and light colored; some little nausea, with occasional vomiting. ℞ Leeches to lower part of abdomen; calomel and opium.

September 1st. Pulse more frequent, more volume; pain over the eyes; skin dry and hot; bowels still loose. Bled to 16 ounces. Calomel and opium repeated.

2d. Some better; pulse reduced in force and frequency; skin moist; no pains; discharges from bowels more frequent, but of dark bilious color; no abdominal tenderness. ℞ Blister to lower part of abdomen, calomel and quinine ää grs. x. morph. grs. ii, to be given every three hours. Under this prescription the patient for two days seemed to improve; the discharges from the bowels being less frequent. On the third day the discharges became much more frequent and tinged with blood. The morphia was now increased to four grains every two hours without at all altering the condition of the bowels or procuring a moment's sleep. Mercurial inunction had been resorted to but without producing any sensible effect. Mustard poultices to the extremities. Carb. ammonia, wine whey, and brandy toddy were all given.

Died on the 8th; intellect clear to the last. No post-mortem examination.

Case III.—J. P., æt. 30, tall, not very muscular, ship carpenter, habits good, lived in same room with case 2d; taken sick at the same time, and with symptoms so analogous, that one prescription answered for both. This almost identity of symptoms, and consequently of treatment continued until the fourth day, when in case 3d a profuse hemorrhage from the bowels (two quarts, according to the nurse's estimate) occurred; the patient was much prostrated by this discharge of blood. A little brandy toddy was given occasionally for six or eight hours, when quinine and morphia in very small doses were prescribed, and continued several days. On the eighth day discharged half a pint of blood from the nose. Discontinued attendance on the tenth day; convalescence dating from the first hæmorrhage.

*Remarks.*—In the three cases here reported we have a type of fever prevailing, more or less in the latter part of every summer and fall, in Louisville and its vicinity. The particular locality in which these cases occurred (near the ship yard) is especially subject to this form of disease. Two causes may be assigned for this,—1st. The situation is low and flat, subject very rarely to overflow, but after every rain remaining very damp, with several large ponds of water upon it for weeks; this low land extends half a mile back of the ship yard to Beargrass creek, which empties into the Ohio river at the city steamboat landing. Unless the season be unusually wet, this creek is little else than a chain of stagnant ponds, into which much filth is thrown from tan-yards and slaughter houses. 2dly. The very great increase in the boat building of this city, and the consequent demand for ship carpenters, attracts every spring a large number of men from the most healthy portions of Ohio, Pennsylvania, New York, &c., and who of course are entirely unused to our climate. Cases 1st and 2d were from Elizabethtown, Pennsylvania; and case 3d from Maine. This disease, however, is by no means confined to this locality; it occurs occasionally in the densely populated part of the city—in Jeffersonville, Indiana, on the opposite bank of the river, and is very common indeed in the surrounding counties.

Does it differ at all from the ordinary autumnal remittent fever, as it has prevailed throughout the West and South for the last few years, or the last half century, at particular seasons? Let the readers of the Western Journal answer.

I have thought that a detailed account of a few cases might be instructive as throwing some light, not upon the cause of fever perhaps, but upon those structural lesions which are the immediate causes of death, and thereby leading to a more rational and more successful treatment. The most interesting points in their histories, are the entire absence of pain upon pressure over the abdomen in one, and the very slight pain in the other two, notwithstanding the intense and extensive inflammation in the organs of this cavity; and the little influence that opium and its preparations had in controlling the discharges from the bowels. That the same lesions were present in the two last cases, which dissection revealed in the first, there can be no doubt; the close analogy between the symptoms in all these cases, and the identical effects of the same remedies, would alone be sufficient to prove it; but again, in case second there was slight hemorrhage from the bowels, and in case third it was most profuse, prostrating the patient very much, and caused in all probability by the sudden rupture of a blood vessel from ulceration. What practical deductions are to be drawn from these cases? 1st. That absence of pain or tenderness upon pressure over the abdomen, is not inconsistent with the most extensive inflammation and ulceration of the bowels. 2d. That as in case third a decidedly favorable change dated from a very copious hemorrhage, when the pulse did not seem at all to indicate the loss of blood; threfore free depletion, either general or local, would promise benefit in such cases, and the pulse cannot be relied upon as an infallible guide. 3d. That the liver is healthy, secreting bile with its usual energy, and as sensible to the action of its appropriate stimulus, mercury, as in perfect health; that therefore large doses of calomel for the purpose of acting upon this organ are not indicated, and productive of no benefit. 4th. That we cannot hope by the largest doses of opium and its preparations to arrest the discharges from the bowels until by depletion the inflammation is relieved.

Louisville, December, 1845.

